# Design of Selective Gas Sensors Using Additive-Loaded In_2_O_3_ Hollow Spheres Prepared by Combinatorial Hydrothermal Reactions

**DOI:** 10.3390/s111110603

**Published:** 2011-11-07

**Authors:** Sun-Jung Kim, In-Sung Hwang, Yun Chan Kang, Jong-Heun Lee

**Affiliations:** 1 Department of Materials Science and Engineering, Korea University, Seoul 136-713, Korea; E-Mails: raosun@korea.ac.kr (S.-J.K.); herdreamform@korea.ac.kr (I.-S.H.); 2 Department of Chemical Engineering, Konkuk University, Seoul 143-701, Korea; E-Mail: yckang@konkuk.ac.kr

**Keywords:** gas sensor, In_2_O_3_ hollow spheres, combinatorial method, selective detection, pattern recognition

## Abstract

A combinatorial hydrothermal reaction has been used to prepare pure and additive (Sb, Cu, Nb, Pd, and Ni)-loaded In_2_O_3_ hollow spheres for gas sensor applications. The operation of Pd- and Cu-loaded In_2_O_3_ sensors at 371 °C leads to selective H_2_S detection. Selective detection of CO and NH_3_ was achieved by the Ni-In_2_O_3_ sensor at sensing temperatures of 371 and 440 °C, respectively. The gas responses of six different sensors to NH_3_, H_2_S, H_2_, CO and CH_4_ produced unique gas sensing patterns that can be used for the artificial recognition of these gases.

## 1. Introduction

Chemoresistive n-type oxide semiconductors such as SnO_2_, ZnO, TiO_2_, In_2_O_3_, and WO_3_ have been widely used to detect explosive, toxic and harmful gases [1,2]. The main advantages of oxide semiconductor sensors are the simple and cost-effective detection of various gases. High gas responses for detecting trace concentrations of analyte gases can be accomplished by employing well-defined nanostructures [3]. However, selective gas detection using oxide semiconductor sensors is often difficult because a number of different reducing gases can interact electrochemically with negatively charged surface oxygen. Various approaches have been employed to enhance the selectivity of sensors, which include the manipulation of sensing temperature [4,5], the addition of noble metal and oxide catalysts [6,7], coating with a catalytic filtering layer [8], compositional control of composite sensing materials [9], and the use of a neural network algorithm [10].

Combinatorial chemistry provides an attractive and promising approach for high-throughput screening of medicine, catalysts, and functional materials [11–14]. Generally, the combinatorial methods usually use parallel synthesis or characterization for high-speed screening. However, combinatorial approaches can be also applied to the compositional design of composite materials. Accordingly, most of the approaches to achieve highly selective gas sensors through the compositional control of sensing materials, catalysts and additives can be best optimized using combinatorial approaches [15–17]. Moreover, abundant gas sensing characteristics attained by combinatorial investigation can be used as a valuable gas sensing library for the discrimination of complex chemical quantities via the pattern recognition mechanism. Several recent researches have verified the potential of combinatorial approaches for the development of high performance gas sensors [18–22].

Hollow structures are promising nanoarchitectures for the applications of gas sensors on account of their high surface area and gas accessible configurations of thin shells [23,24]. Not only the outer surfaces but also the inner ones participate in the gas sensing reaction. In general, oxide hollow structures are prepared by applying a coating of metal precursors onto polymeric spheres and subsequent removal of sacrificial templates by heat treatment [25,26]. Among various template-based synthetic routes, hydrothermal reaction of a solution containing a metal precursor and glucose or sucrose provides a simple, one-pot method to prepare metal-precursor-coated carbon spheres [27,28]. Hydrothermal condensation of glucose or sucrose into carbon spheres with hydrophilic surfaces [29] enables the uniform coating of metal precursors [27]. Indeed, oxide hollow structures prepared by glucose- or sucrose-mediated hydrothermal reaction showed high gas responses [28,30].

In this contribution, various metal or metal oxide additives are loaded onto In_2_O_3_ hollow spheres in a combinatorial manner by one-pot hydrothermal reaction of a solution containing glucose, In-precursors, and additive-precursors with subsequent heat treatment, and the gas responses to CH_4_, NH_3_, H_2_, CO, and H_2_S have been measured. The main focus of the study is directed at the high-throughput screening of selective gas sensors by combinatorial control of oxide additives and sensor temperatures.

## 2. Experimental Section

Indium (III) nitrate hydrate [In(NO_3_)_3_·xH_2_O, 99.9% metal basis, Sigma-Aldrich, Co.], copper (II) chloride dehydrate (CuCl_2_·2H_2_O, 99% Cica-reagent, Kanto Chem. Co.), niobium (V) pentachloride (NbCl_5_, 99%, Sigma-Aldrich, Co.), nickel (II) chloride hexahydrate (NiCl_2_·6H_2_O, 99.9%, Sigma-Aldrich, Co.), palladium (II) chloride (PdCl_2_, 99%, Sigma-Aldrich, Co.), antimony (III) chloride (SbCl_3_, 98%, Kanto Chem. Co.) and d-(+)-glucose monohydrate (C_6_H_12_O_6_·H_2_O, 99.5%, Sigma-Aldrich, Co.) were purchased and used without further purification.

Pure and additive-loaded In_2_O_3_ hollow spheres were prepared by glucose-mediated hydrothermal reaction. d-(+)-Glucose monohydrate (5.9451 g) was dissolved in distilled water (60 mL). Subsequently, indium (III) nitrate hydrate (0.6017 g) was dissolved and stirred for 15 min. This solution was used for the preparation of the pure In_2_O_3_ hollow spheres. For the preparation of additive-loaded In_2_O_3_ hollow spheres, the corresponding amount (1 wt% compared to In_2_O_3_) of additive source was added to the above solution. These stock solutions were transferred into a Teflon-lined stainless steel autoclave, which was then sealed and heated at 180 °C for 24 h. After hydrothermal reaction, the product was washed with distilled water 4 times and ethanol 1 time by centrifuge and dried at 70 °C for 24 h. The pure and additive-loaded In_2_O_3_ hollow spheres could be prepared by the heat treatment of the above products at 500 °C for 2 h. For simplicity, hereinafter, the pure, Cu, Nb, Ni, Pd, Sb-loaded In_2_O_3_ hollow spheres after heat treatment will be referred as In_2_O_3_, Cu-In_2_O_3_, Nb-In_2_O_3_, Pd-In_2_O_3_, Ni-In_2_O_3_, and Sb-In_2_O_3_ specimens, respectively. The morphologies of the hollow spheres were analyzed by field-emission scanning electron microscopy (FE-SEM, S-4800, Hitachi Co. Ltd.).

For the gas sensing measurement, 0.1 g of each prepared hollow sphere was dispersed in 10 mL of D.I. water and these solutions were deposited on the sensor substrate by using the drop-coating technique. An alumina substrate (1.5 × 1.5 mm^2^) with two Au electrodes on its top surface and a micro-heater on its bottom surface was used. The temperature of the sensors was controlled by modulating the power of the microheater underneath the substrate. The sensor temperature was measured to be 371 and 440 °C at the heater powers of 400 and 500 mW, respectively, by an IR temperature sensor (Rayomatic 14814-2, Euroton IRtec Co.). The uncertainty of sensor temperature was ±5 °C. The sensor was positioned in a specially designed quartz tube chamber and dry synthetic air and mixing gas were flowed into this chamber. The gas response (*S = R_a_/R_g_*, *R_a_*: resistance in air, *R_g_*: resistance in gas) to 500 ppm CH_4_, 100 ppm NH_3_, H_2_, CO, and 5 ppm of H_2_S were measured using a multimeter (Keithley K2000) which connected with a computer.

## 3. Results and Discussion

**Figure 1.**
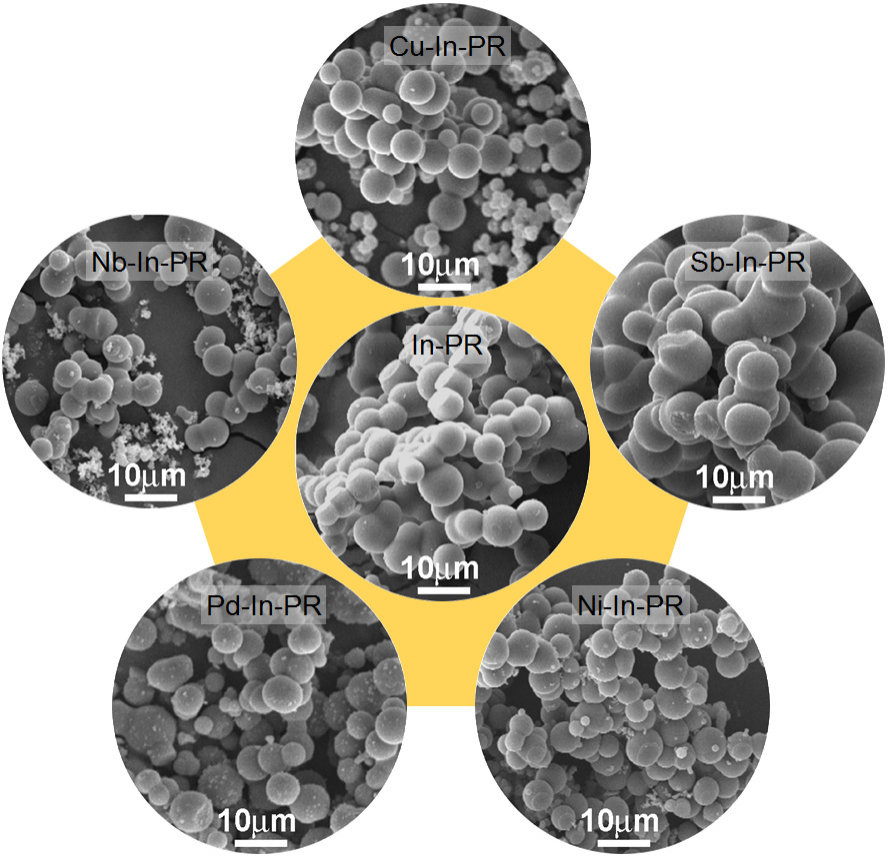
SEM images of as-prepared carbon spheres coated with In- and additive-precursors: In-PR (precursor spheres to be converted into In_2_O_3_ hollow spheres); M-In-PR (M = Sb, Cu, Nb, Pd, and Ni; precursor spheres to be converted into M-In_2_O_3_ hollow spheres).

**Figure 2.**
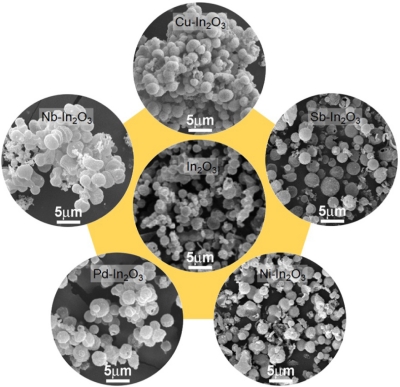
SEM images of In_2_O_3_, Sb-In_2_O_3_, Cu-In_2_O_3_, Nb-In_2_O_3_, Pd-In_2_O_3_ and Ni-In_2_O_3_ spheres after heat treatment at 500 °C for 2 h.

All the as-prepared specimens after hydrothermal reaction were spheres with a size of 5–7 μm (Figure 1). The surface morphology, the presence of nano-size particles, and the connectivity between carbon spheres were slightly different for each specimen according to the doping of additives. After heat treatment of the precursor spheres at 500 °C for 2 h, the as-prepared precursor spheres with clean surfaces (Figure 1) were converted into spheres with rough surfaces consisting of primary nanoparticles (Figure 2).

The average diameters of ∼100 In_2_O_3_, Sb-In_2_O_3_, Cu-In_2_O_3_, Nb-In_2_O_3_, Pd-In_2_O_3_ and Ni-In_2_O_3_ spheres were 2.3 ± 0.5 μm, 2.4 ± 0.7 μm, 2.2 ± 0.4 μm, 2.3 ± 0.6 μm, 2.3 ± 0.5 μm, and 2.2 ± 0.5 μm, respectively. The decrease of sphere diameters during heat treatment can be attributed to the shrinkage of spheres by the decomposition of carbon cores.

**Figure 3.**
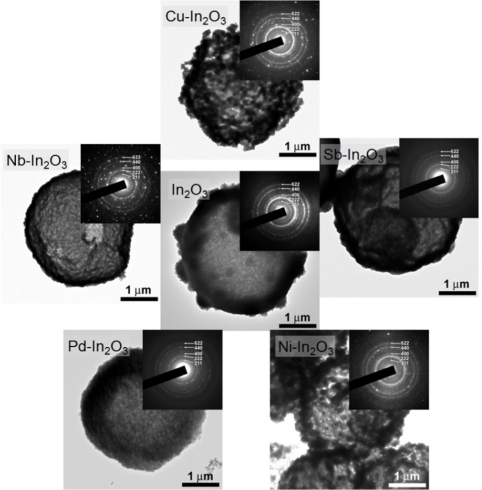
TEM images and selected area electron diffraction patterns of In_2_O_3_, Sb-In_2_O_3_, Cu-In_2_O_3_, Nb-In_2_O_3_, Pd-In_2_O_3_ and Ni-In_2_O_3_ hollow spheres after heat treatment at 500 °C for 2 h.

Figure 3 shows the TEM images of the specimens after heat treatment at 500 °C for 2 h. All the specimens showed bright contours in the centers of spheres, which indicated the hollow morphology. The selected area electron diffraction patterns of hollow spheres were indexed as cubic In_2_O_3_ phases. The thicknesses of shells depended on the additives, which ranged from 50 nm to 200 nm.

**Figure 4.**
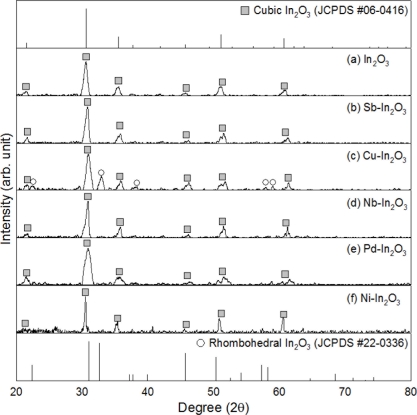
X-ray diffraction patterns of (a) In_2_O_3_, (b) Sb-In_2_O_3_, (c) Cu-In_2_O_3_, (d) Nb-In_2_O_3_, (e) Pd-In_2_O_3_ and (f) Ni-In_2_O_3_ hollow spheres after heat treatment at 500 °C for 2 h.

The In_2_O_3_, Nb-In_2_O_3_, Ni-In_2_O_3_, Pd-In_2_O_3_ and Sb-In_2_O_3_ specimens after heat treatment at 500 °C for 2 h were identified as pure cubic In_2_O_3_ phase (JCPDS# 06-0416) by X-ray diffraction (XRD) (Figure 4).

The analyses of possible second phases were difficult probably due to the detection limit of XRD. In the Cu-In_2_O_3_ specimen, a small amount of corundum-type rhombohedral phase (JCPDS# 22-0336) co-existed. The rhombohedral phase is known to be stable at high pressure [31]. However, it has been reported that a metastable rhombohedral phase can be prepared by doping with Sn^4+^ or Fe^3+^ [32,33], or by sol-gel based synthesis [34]. Considering the cubic phase of the pure In_2_O_3_ specimen, the rhombohedral structure of the Cu-In_2_O_3_ specimen might be understood as the result of Cu^2+^ incorporation into the lattice of In_2_O_3_, although further systematic studies are necessary to confirm this.

**Figure 5.**
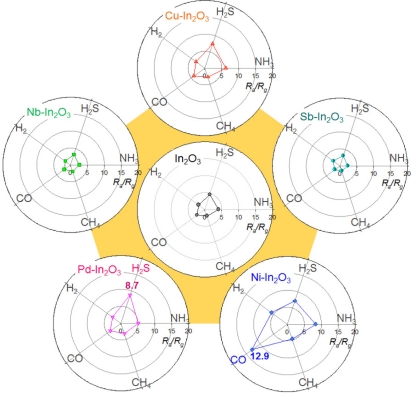
Polar plots of gas responses to 100 ppm NH_3_, 5 ppm H_2_S, 100 ppm H_2_, 100 ppm CO, and 500 ppm CH_4_ of In_2_O_3_, Sb-In_2_O_3_, Cu-In_2_O_3_, Nb-In_2_O_3_, Pd-In_2_O_3_ and Ni-In_2_O_3_ sensors at 371 °C.

At the sensor temperature of 371 °C, the gas responses of the In_2_O_3_ sensor to 100 ppm NH_3_, 5 ppm H_2_S, 100 ppm H_2_, 100 ppm CO and 500 ppm CH_4_ ranged from 2.0 to 4.6 (Figure 5). All the gas responses were decreased by the loading of Nb and Sb. In contrast, the loading of Cu increased the responses to all the gases. In the Cu-In_2_O_3_ sensor, although the response to H_2_S (7.5) was higher than the response to the other 4 gases, it was not markedly higher than the response to NH_3_ (6.2). Among 5 different sensors, the loading of Pd showed the most selective detection of H_2_S. The H_2_S response of the Pd-In_2_O_3_ sensor was 8.7 while the responses to NH_3_, H_2_, CO, and CH_4_ were 5.1, 3.0, 3.9, and 3.2, respectively. The selectivity to a specific gas was defined as “*S_SG_/S_IG_*” (*S_SG_*: gas response to specific gas, *S_IG_*: gas response to interference gas). The *S_H2S_/S_IG_* values ranged from 1.7 to 5.7. The loading of Ni increased all the gas responses. In particular, the response to CO was enhanced to a great extent. The *S_CO_/S_IG_* values ranged from 1.6 to 2.2. Thus, the high response to CO (12.9) with the lower cross-responses to NH_3_, H_2_S, H_2_, and CH_4_ (5.8–8.3) demonstrates that the Ni-In_2_O_3_ sensor can be used for selective CO detection.

**Figure 6.**
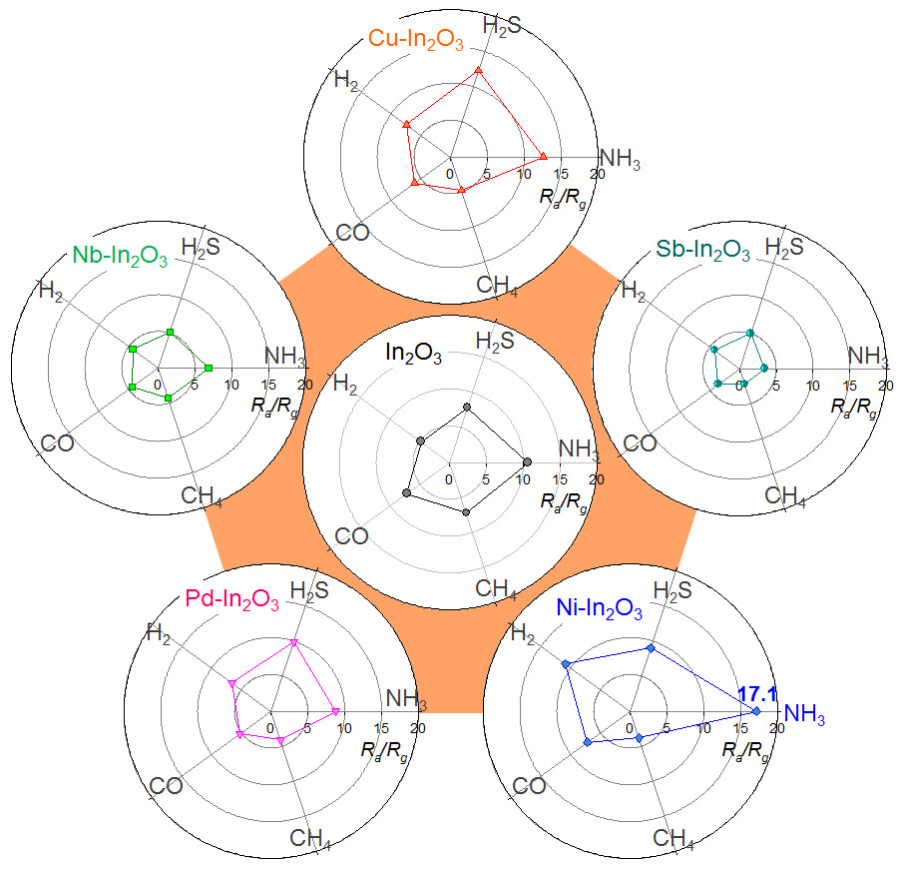
Polar plots of gas responses to 100 ppm NH_3_, 5 ppm H_2_S, 100 ppm H_2_, 100 ppm CO, and 500 ppm CH_4_ of In_2_O_3_, Sb-In_2_O_3_, Cu-In_2_O_3_, Nb-In_2_O_3_, Pd-In_2_O_3_ and Ni-In_2_O_3_ sensors at 440 °C.

The selectivity of the gas sensing reaction was also influenced by the variation of sensor temperature. When the sensor temperature was increased to 440 °C, the gas responses of all the sensors tended to increase (Figure 6). In the pure In_2_O_3_ sensor, the NH_3_ response (10.6) was the highest whereas the response to H_2_S (7.2) was comparable. The loadings of Sb and Nb lead to the decrease of gas responses. The responses to H_2_S and NH_3_ of the Cu-In_2_O_3_ sensor were 12.3 and 12.6, respectively, which were higher than those to H_2_, CO and CH_4_ (4.8–7.4). The responses to H_2_S and NH_3_ of the Pd-In_2_O_3_ sensor (9.9 and 8.8) were also similar to each other. Finally, the response to NH_3_ of the Ni-In_2_O_3_ sensor (17.1) was significantly higher than those to other gases (3.8–10.9) at the sensor temperature of 440 °C. The *S_NH3_/S_IG_* values ranged from 1.6 to 4.5. The above results indicate that the selectivity of the gas sensor is influenced not only by the additives but also by the gas sensing temperature [35].

The Pd-In_2_O_3_ and Cu-In_2_O_3_ sensors showed the most selective H_2_S detection at 371 °C. As the sensor temperature is increased to 440 °C, discrimination between H_2_S and NH_3_ becomes difficult. In the literature, the conversion of p-type CuO into metallic CuS by interaction with H_2_S is regarded as a key reason for the selective H_2_S detection of CuO-loaded SnO_2_ gas sensors [36–38]. The relatively high H_2_S sensitivity of Cu-In_2_O_3_ can be understood from this viewpoint. Pd is a representative noble metal catalyst which is used to enhance the gas sensing characteristics of oxide semiconductors. To date, Pd has been added to SnO_2_, ZnO, In_2_O_3_ and TiO_2_ sensors to improve the sensing characteristics for C_2_H_5_OH [39], NH_3_ [40], LPG [41], CO [42], and H_2_S [43]. The diverse roles of Pd additives in the gas sensing reaction seem to relate to the physico-chemical state, loading concentration, size, and distribution of the Pd catalyst. Zhang *et al.* [43] reported that the loading of Pd nanoparticles on the surface of ZnO nanowires by a self-assembly reaction significantly enhanced both the gas response and selectivity to H_2_S. This is consistent with the present result.

Selective detections of CO and NH_3_ were achieved when the Ni-In_2_O_3_ sensor was operated at 371 °C or 440 °C, respectively (Figures 5 and 6). The effects of NiO loading on the gas sensing characteristics of SnO_2_ are not always consistent in the literature. Both enhancements [44,45] and deteriorations [46,47] of gas responses have been reported. Considering that NiO is a p-type oxide semiconductor, the positive and negative roles of NiO in the gas sensing reaction can be explained by the extension of the electron depletion layer due to the formation of a p-n junction and by the counteraction of resistance variation upon exposure to reducing gases. In the present study, the enhancement of specific gas responses to CO and NH_3_ should be understood in the framework of catalytic promotion of the sensing reaction and/or acid/base interaction between additives and analyte gas.

High gas response to a specific gas with a negligible cross-response is desirable for selective and quantitative gas detection. The present results do not show perfect selectivity. However, the change in the gas sensing pattern by loading of different additives and by controlling sensor temperature can be used to discriminate between the gases by using a pattern recognition algorithm. To date, several combinatorial approaches have been suggested to get different gas sensing patterns [48–52]. Aronova *et al.* [51] reported gas sensing patterns for chloroform, formaldehyde, and benzene using SnO_2_ sensors loaded with various additives such as ZnO, WO_3_, In_2_O_3_, Pt and Pd. Siemons *et al.* [52] achieved the selective detection of CO and NO_2_ by combinatorial loading of 7 different noble metals to La-CoTiO_3_ sensors.

**Figure 7.**
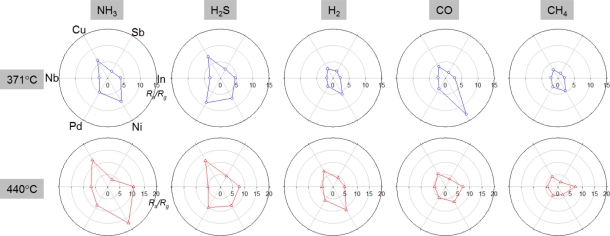
Gas sensing patterns of In_2_O_3_ (In), Sb-In_2_O_3_ (Sb), Cu-In_2_O_3_ (Cu), Nb-In_2_O_3_ (Nb), Pd-In_2_O_3_ (Pd) and Ni-In_2_O_3_ (Ni) sensors to NH_3_, H_2_S, H_2_, CO and CH_4_ at 371 and 440 °C.

The gas sensing patterns using six different sensors were plotted in Figure 7. The relative gas responses in the polar plots showed the unique sensing patterns for each analyte gas. The operation of 6 different sensors at 371 °C can distinguish between CO and H_2_S. Characteristic sensing patterns of NH_3_, H_2_S, and CH_4_ at 440 °C facilitate gas discrimination via a pattern recognition algorithm. When this sensor array is used to detect a specific gas in real applications, the interferences from the cross-responses to other gases should be minimized by optimizing the pattern recognition algorithm considering the operation environment. The loading of various metal and metal oxide additives to In_2_O_3_ hollow spheres via the combinatorial hydrothermal route is a high-throughput approach to screening the highly selective gas sensors and to distinguishing a gas by using a pattern recognition algorithm.

## 4. Conclusions

Hollow spheres of pure In_2_O_3_ and Sb-, Cu-, Nb-, Pd-, and Ni-loaded In_2_O_3_ were prepared by combinatorial hydrothermal reaction of a solution containing glucose, In-precursor and additive-precursor with subsequent heat treatment. The selective detections of H_2_S, CO, and NH_3_ were achieved by the control of additives and sensing temperatures. The sensing patterns at 371 and 440 °C using six different sensors provided the characteristic signal patterns that were sufficient to discriminate between CO, H_2_S, NH_3_, H_2_S, and CH_4_. Combinatorial design of additive-loaded In_2_O_3_ hollow spheres facilitates high-throughput screening of selective gas sensors as well as the discrimination of gases via pattern recognition.
